# Exposure to Famine During Early Life and Abdominal Obesity in Adulthood: Findings from the Great Chinese Famine During 1959–1961

**DOI:** 10.3390/nu11040903

**Published:** 2019-04-22

**Authors:** Dan Liu, Dong-mei Yu, Li-yun Zhao, Hong-yun Fang, Jian Zhang, Jing-zhong Wang, Zhen-yu Yang, Wen-hua Zhao

**Affiliations:** National Institute for Nutrition and Health, Chinese Center for Disease Control and Prevention, 27 Nanwei Road, Xicheng District, Beijing 100050, China; liudanjulie@163.com (D.L.); yudm@ninh.chinacdc.cn (D.-m.Y.); zhaoly@ninh.chinacdc.cn (L.-y.Z.); fanghy@ninh.chinacdc.cn (H.-y.F.); zhangjian@ninh.chinacdc.cn (J.Z.); wangjz@ninh.chinacdc.cn (J.-z.W.); yangzy@ninh.chinacdc.cn (Z.-y.Y.)

**Keywords:** undernutrition, abdominal obesity, fetal, infant, adulthood

## Abstract

Undernutrition during early life may lead to obesity in adulthood. This study was conducted to examine the relationship between famine exposure during early life and the risk of abdominal obesity in adulthood. A total of 18,984 and 16,594 adults were surveyed in 2002 and 2010–2012 in two nationally representative cross-sectional surveys, namely China Nutrition and Health Survey, respectively. The risk of abdominal obesity was evaluated for participants born during 1956–1961 and compared with that of participants born during 1962–1964. The overall prevalence of abdominal obesity in adulthood showed a positive association with famine exposure during early life. The odds ratios of famine exposure were 1.31 (1.19–1.44) and 1.28 (1.17–1.40) in 2002 during fetal life and infancy and 1.09 (1.00–1.19) in 2012 during fetal life, respectively. The relationships between famine exposure and abdominal obesity across the famine exposure groups were distinct among females and those who lived in urban areas and were physical inactive (*p* < 0.05). Exposure to famine during early life was associated with increased risks of abdominal obesity in adulthood, which was partially alleviated by healthy lifestyle factors (e.g., physical activity).

## 1. Introduction

Overweight and obesity, as well as abdominal obesity, in middle age are strongly related to all-cause mortality and morbidity of chronic diseases such as diabetes and hypertension and other metabolic diseases [1,2]. Several risk factors have been considered to be responsible for the development of systemic obesity and abdominal obesity, such as diet, lifestyle, and genetic background [3]. The Developmental Origins of Health and Disease hypothesis suggests that [4,5,6] undernutrition during early life may be associated with obesity. Nutritional status during critical window periods of early life may have long-lasting effects on health in adulthood. Famine study is a commonly used approach to test the hypothesis in humans. Undernutrition is considered as natural exposure in a famine. For example, the Dutch ”hungry winter” famine and the Great Chinese Famine were used to evaluate the “fetal origins” hypothesis [7]. 

Previous studies have primarily focused on the relationship between famine and body mass index (BMI) and/or systemic obesity [8,9,10,11,12]. The relationship between early-life famine exposure and waist circumference and/or abdominal obesity in later life has been less studied. Abdominal obesity is a well-established risk factor for metabolic diseases, independent of BMI [13], and could influence the risk for disease through increased insulin resistance [14]. Therefore, it is important to explore the risk factors of abdominal obesity. The Great Chinese Famine is an opportunity to evaluate the correlations between famine exposure during early life and abdominal obesity in adulthood.

The Great Chinese Famine that occurred during 1959–1961 is one of the most disastrous catastrophes in human history, resulting in 20–30 million deaths [15,16,17] throughout China. The Dutch famine occurred within a well-nourished population and may have led to less severe effects on human health [18]. In contrast, the Great Chinese Famine had longer duration, and food availability was more severely curtailed nationwide. Thus, a greater impact of the Great Chinese Famine on adult health can be expected than that of the Dutch famine. Therefore, national studies on the Great Chinese Famine are good resources for investigating the relationship between famine and the subsequent effects of malnutrition in early years on abdominal obesity and other chronic diseases and also for assessing whether adult lifestyle would alter the effect of the famine.

Two nationally representative cross-sectional surveys were conducted 40 and 50 years after the Great Chinese Famine in 2002 and 2010–2012 in China, respectively. These surveys could be good sources for analyzing the relationship between famine and abdominal obesity in participants of middle age and pre-elder age. 

## 2. Participants and Methods

### 2.1. Design and Participants

Data were extracted from the China Nutrition and Health Survey (CNHS), a nationally representative cross-sectional study on nutrition and chronic diseases. A stratified, multistage probability cluster sampling design was used in this survey, which has been described in detail previously [19]. In the present study, data from two surveys of CNHS conducted in 2002 and 2010–2012 were used to evaluate the long-lasting impact of famine on abdominal obesity, 40 and 50 years after the occurrence of the Great Chinese Famine, respectively. The study population consisted of participants with date of birth (DOB) between October 1, 1956, and September 30, 1964. To minimize misclassification of the exposure periods, participants with DOB between October 1, 1958, and September 30, 1959, and between October 1, 1961, and September 30, 1962, were excluded because the exact dates of the start and the end of the Chinese famine were not available in different regions. The total sample size was 18,984 adults from CNHS 2002 and 16,594 adults from CNHS 2010–2012. All procedures involving participants were approved by the Medical Ethics Committee at the National Institute for Nutrition and Health, Chinese Center for Disease Control and Prevention. All participants provided their written informed consent.

### 2.2. Famine Exposure

Participants were categorized into the following three predefined groups according to their DOB: (1) nonexposed, with DOB between October 1, 1962, and September 30, 1964, (2) fetal-exposed, with DOB between October 1, 1959, and September 30, 1961, and (3) infant-exposed, with DOB between October 1, 1956, and September 30, 1958. Mean ages of the participants in these three exposed groups were respectively 38.6, 41.6, and 44.6 years in 2002 and 48.2, 51.2, and 54.1 years in 2010–2012.

Although the Great Chinese Famine affected the entire mainland of China, its severity varied across provinces due to different weather conditions, population density, and local policies pertaining to food shortage [20]. The severity of the famine was determined based on the excess death rate (EDR) of each province [20]. Participants were classified into severe famine exposure group and moderate famine exposure group based on residential provinces after excluding participants without local permanent residency. The EDR was calculated as the percentage change in mortality rate from the mean level in 1956–1958 to the highest value during the period 1959–1961 [20]. The median of the EDR was used as the cutoff point, which was consistent with other studies [21]. Provinces with an EDR equal to or above the cutoff point were categorized as severe famine exposure areas, and otherwise as moderate famine exposure areas.

### 2.3. Anthropometric Measurements

Anthropometric measurements included body weight, height, and waist circumference. Height was measured using a stadiometer (model no. SG-210, Nantong yue kin cervix equipment Co., Ltd, Nantong, China) after removing shoes, and body weight was measured with light clothes using a beam scale (model no. RGT-14-RT, Wuxi Weighing Factory Co., Ltd, Wuxi, China). Waist circumference was measured using a waist circumference tape (model no.0403, Nanjing Kongki Commodity Co., Ltd, Nanjing, China) only after breathing out. The accuracy of the height, waist circumference, and weight measurements was 0.1 cm, 0.1 cm, and 0.1 kg, respectively. The anthropometric measurements were made according to standard anthropometric measurement methods in health surveillance [22]. All anthropometric measurement staff were trained according to the standard procedure. Standardized tests were conducted for all trainees, and only those being trained and passing an examination were given a qualification certificate for conducting anthropometric measurements. Each anthropometric measurement staff was retested partially to ensure the inter-rater reliability for each anthropometric measurement. Height and weight were used to calculate BMI, by dividing weight (kg) by height squared (m^2^). Systemic obesity and abdominal obesity were defined using the Chinese criteria of weight for adults [23]. Systemic obesity was defined as BMI ≥28 kg/m^2^. Overweight was defined as BMI ≥24 and <28 kg/m^2^. Abdominal obesity was defined as waist circumference >90 cm in men and ≥85 cm in women.

### 2.4. Covariates

Residential areas were classified into urban and rural. Physical activity was categorized into active and inactive level, wherein regular exercise for >20 min each time, including various activities such as running, swimming, and performing Tai Chi, was defined as active level. Current drinking status and current smoking status were considered as dichotomous variables based on the answer in the questionnaires (yes or no). Current drinking was defined as participants drinking alcohol in the past year, irrespective of the amount drunk. Current smoking was defined as smoking regularly every day or irregularly. Education level was categorized into dichotomous variable, where high school or above was considered as high educational level, otherwise as low educational level.

### 2.5. Statistical Analyses

The SAS version 9.4 (SAS Institute Inc., Cary, NC, USA) was used for all statistical analyses, and a two-sided *p* value <0.05 was considered to be statistically significant. Odds of abdominal obesity for the fetal-exposed group and the infant-exposed group, compared with the nonexposed group, were examined by the maximum likelihood method using the logistic regression model. Analyses were adjusted for sex, residential areas, education level, marital status, household income, current drinking status, current smoking status, and physical activity. To investigate whether the associations between fetal and infant exposure to famine and abdominal obesity were affected by social environment in later life, we subsequently stratified the analyses according to sex, residential areas, physical activity, and education level. The odds ratios (95% confidence interval (CI)) of abdominal obesity in the fetal- and infant-exposed groups compared with the nonexposed group were calculated within each category of the stratified variables. The stratified variables were not adjusted in the corresponding models. The odds ratios (95% CI) were plotted in a graph using Stata 13.0. Sensitivity analysis was also performed in this study. First, we selected the 75th percentile of EDR as the cutoff point, and an EDR of 150.0% was used to define the severity of famine for the purpose of distinguishing severely and moderately severely affected famine areas more significantly. Second, participants with BMI ≥28.0 kg/m^2^ who were categorized as having systemic obesity according to the Chinese criteria were excluded to prevent the interaction between systemic obesity and abdominal obesity.

## 3. Results

Table 1 lists the basic characteristics of participants according to famine exposure. A total of 4352 (22.9%) and 6469 (34.1%) participants in CNHS 2002 and 4126 (24.9%) and 5975 (36.0%) participants in CNHS 2010–2012 were exposed to the Great Chinese Famine during their fetal and infant period, respectively. Compared with the nonexposed group, participants in the fetal-exposed group had a significantly greater waist circumference in both 2002 and 2010–2012 (both *p* < 0.05 after Bonferroni correction), whereas the infant-exposed group showed a greater waist circumference only in 2002. The prevalence rates of abdominal obesity in nonexposed, fetal-exposed, and infant-exposed groups were 15.5%, 19.4%, and 18.9% in 2002 and 31.6%, 33.3%, and 32.6% in 2010–2012, respectively.

Table 2 shows the associations of famine exposure with abdominal obesity risk and the stratified analysis according to famine severity. In general, the prevalence of abdominal obesity in 2010–2012 was higher than that in 2002 in each group. Compared with the nonexposed group (1962.10–1964.9), participants had a significantly higher prevalence of abdominal obesity in both 2002 and 2010–2012 with an odds ratio (95% CI) of 1.31 (1.19–1.44) and 1.09 (1.00–1.19) in the fetal-exposed group (1959.10–1961.9) and only in 2002 with an odds ratio (95% CI) of 1.28 (1.17–1.40) in the infant-exposed group (1956.10–1958.9). After stratification of the study areas according to famine severity, participants showed a significantly higher prevalence of abdominal obesity in severely affected famine areas in both 2002 and 2010–2012 with an odds ratio (95% CI) of 1.32 (1.14–1.52) and 1.13 (1.01–1.27) in the fetal-exposed group; in the infant-exposed group, the higher prevalence of abdominal obesity was statistically significant only in 2002 compared with that in the nonexposed group. All odds ratios were adjusted for sex, residential areas, education level, marital status, household income, current drinking status, current smoking status, and physical activity. 

Table 3 shows the results of the sensitivity analysis. The severely affected famine areas were defined as those with an EDR ≥150% (sensitivity analyses A). Participants who were born in severely affected famine areas had a significantly higher prevalence of abdominal obesity in both 2002 and 2010–2012 in the fetal–exposed group. In the infant-exposed group, the higher prevalence of abdominal obesity was statistically significant only in 2002.

In addition, participants with BMI ≥28 kg/m^2^ were excluded in sensitivity analyses B. The results of the fetal-exposed group were similar to those in sensitivity analyses A. After excluding the effect of systemic obesity, the rate of abdominal obesity was higher in the severely affected famine areas than that in the moderately severely affected famine areas in 2010–2012.

**Figure 1:**
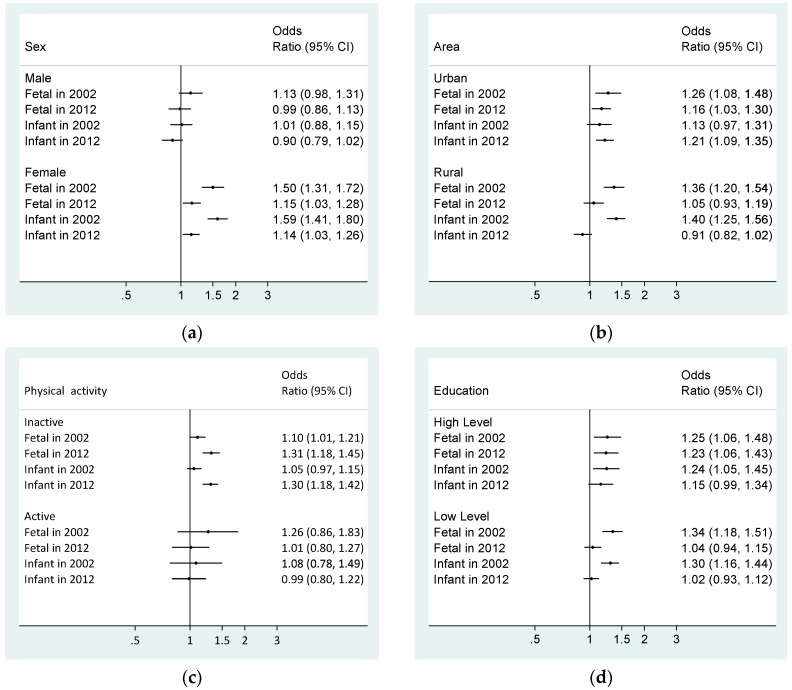
Subgroup analyses of the association between central obesity and famine exposure groups. (**a**) Associations of famine exposure with central obesity risk by sex; (**b**) Associations of famine exposure with central obesity risk by area; (**c**) Associations of famine exposure with central obesity risk by physical activity; (**d**) Associations of famine exposure with central obesity risk by education level. Model adjusted for sex, residential areas, education level, marital status, household income, current drinking status, current smoking status, and physical activity. Stratified variables were not adjusted in the corresponding models. CI, confidence interval. All odds ratios used the nonexposed group as the reference.

In CNHS 2002 and CNHS 2010–2012, the risk of famine exposure associated with abdominal obesity was detected among female, inactive participants, those who lived in urban areas, and those with high level of education (Figure 1). The results were adjusted for sex, residential areas, education level, marital status, household income, current drinking status, current smoking status, and physical activity. 

## 4. Discussion

Based on two nationally representative studies, the present investigation found that exposure to the Great Chinese Famine during early life increased the risk of abdominal obesity in later adulthood, especially among female, inactive participants, those who lived in urban areas, and those with high education level. The risk of abdominal obesity had a longer lasting effect on those who experienced the Great Chinese Famine in their fetal period. Current unhealthy lifestyle factors would exacerbate the effect of early-life exposure to famine on abdominal obesity.

Low birth weight has been considered to be a risk factor for obesity in adults, which indicated that exposure to famine during early life might increase the risk of obesity in adulthood [8,9]. Some studies reported that fetal or infant exposure to famine could reduce the risk of systemic obesity in adulthood [10,11]. In addition, a few studies have reported that there was no association between famine exposure during some stages of early life and later obesity [12]. However, these previous studies were different in terms of the duration of famine exposure and famine severity, and the recruitment periods were also inconsistent. For example, compared with the Dutch famine (one winter), the Chinese famine lasted longer (3 years) and was more severe. Furthermore, abdominal obesity was more harmful to metabolic diseases than systemic obesity. Because no study had evaluated the association between Chinese early-life famine exposure and abdominal obesity in adulthood during different life stages, data from CNHS 2002 and 2012 were an opportunity to fill the gap. 

The fetal origin of adult disease hypothesis, also referred to as the Barker hypothesis, proposed that alterations in fetal nutrition and endocrine status lead to developmental adaptations that permanently change the body structure, physiology, and metabolism, thereby predisposing individuals to cardiovascular, metabolic, and endocrine diseases in adult life [4,5,6]. Several precise mechanisms might explain the associations between fetal famine exposure and risk of abdominal obesity in later life. First, early-life malnutrition might alter the neuroendocrine function, including induction of the hypothalamic–pituitary–adrenal cortical axis, which results in excessive secretion of glucocorticoids and fat accumulation in later life [24,25]. Second, a probable mechanism is that nutritional deprivation in early life affects the expression of related genes and would change the dietary behavior. Evidence from the Dutch famine study has suggested that prenatal exposure to famine would increase the preference for fatty food and might contribute to more atherogenic lipid profiles in later life, such as belly fat [26]. Third, findings from epigenetic studies have suggested that early malnutrition could lead to abnormal DNA methylation of genes, which was associated with obesity and insulin resistance in adulthood [27]. The above mentioned abnormalities could result in excessive fat deposition. Furthermore, hypertension, diabetes, and coronary heart disease were found to be associated with early-life malnutrition, indicating a relationship between famine exposure and obesity in adulthood [28]. 

The adaptive sex ratio adjustment hypothesis suggested that mothers who experience nutritional stress would be more likely to give birth to female babies. This is because nutrition cost was less expensive for female babies than for male babies, and female babies had a better opportunity to survive in the harsh environment [29]. Male survivors may have “acceptable” nutrition exposure during early life because vulnerable male babies were difficult to survive. On the other hand, male survivors may also be associated with the culture of son preference in China [30]. Famine might predispose female survivors to the risk of developing chronic degenerative diseases in adulthood, including obesity. Evidence has shown that undernutrition during early life had larger long-term impacts on females than on males [24,31,32]. Consistent with these findings, our study has also demonstrated a higher prevalence of abdominal obesity among female survivors in the Great Chinese Famine.

A mismatch between early life and adult life environment may explain the association between famine exposure and risk of abdominal obesity [33,34,35]. Nutrition restriction during early life and exposure to a “rich” environment (rich nutrition, high socioeconomic status, etc.) in later life might increase an individual’s susceptibility or risk of developing obesity and other chronic diseases. Data from the CNHS 2002 showed that modern diet exacerbated the effects of famine in relation to diabetes and hypertension [21,36]. In the present study, participants who lived in urban areas or had a high education level had an increased risk for abdominal obesity across fetal and infant famine exposure groups, which might also be attributed to the modernized life. It has been suggested that people who lived in urban areas and had a high education level were more likely to have a high-energy-dense western dietary pattern in China [19,37,38]. Furthermore, the present study results have demonstrated that the adverse effects of undernutrition during early life were likely to be exacerbated by unhealthy lifestyle factors during later adulthood. Unhealthy lifestyle in the fetal- and infant-exposed groups, including inadequate levels of physical activity, imposed an increased risk of abdominal obesity compared to that in the nonexposed group. These findings further highlighted the importance of a healthy lifestyle in the prevention of adult chronic diseases.

The association between famine exposure and abdominal obesity disappeared in the 2010–2012 survey, such as in rural participants and those with low education level. The significant association existed for the female participants, but the odds ratio became smaller. Overall, the association was attenuated among participants, except for physically inactive participants in 2010–2012. This might be related to the trajectory of the human body shape over the course of the lives. Zhai et al [1] showed that the waist circumference of Chinese adults increased gradually during early age, whereas it began to decline with aging. The trajectory was slightly different between gender, wherein males began to develop a thinner body shape in their 40s and females did so in their 50s. This phenomenon could explain why the association disappeared among males in their 50s but remained in the females. The trajectory of the human body shape could also explain the change in other subgroups, such as the residential area and the education group. The risk of abdominal obesity in participants living in urban areas and having a high education level was still steady and might be related to their exposure to a “rich” environment in later life. However, rural participants with a low education level showed a declining trajectory of waist circumference in the absence of exposure to a “rich” environment.

This study has some limitations. First, the lack of birth weight data might be a concern. However, fetal programming could also occur without any marked effects on birth size [39], and hence this was not considered as a major limitation. Second, the Chinese famine affected almost the entire country. Therefore, participants had to be classified into different groups based on their birth date rather than exposure areas or nonexposure areas. Despite these limitations, our research also had irreplaceable advantages. The Great Chinese Famine lasted much longer and affected more people than other famines. This study could also demonstrate further convincing results. Data from two large, nationally representative cross-sectional surveys conducted 40 and 50 years later in 2002 and 2010–2012 after the Chinese famine were used in this study along with detailed information regarding sociodemographic characteristics, lifestyle factors, and birthplace. Therefore, our research provides valuable evidence on the hypothesis of the combined association between early-life famine exposure and later life environment and the risk of abdominal obesity in adulthood. In addition, undernutrition during early life was associated with metabolic diseases, including metabolic syndrome, diabetes mellitus, and stroke, which would occur around the age of 60 years. In future, more studies investigating the abovementioned diseases are warranted. Furthermore, China might face a high incidence of metabolic diseases in the forthcoming years.

## 5. Conclusions

Early-life exposure to the Great Chinese Famine exacerbated the risk of abdominal obesity, especially in females or those who lived in urban areas or were physically inactive. A healthy lifestyle might partially alleviate the adverse effects. The results suggest that promoting a healthy lifestyle should be considered as a critical strategy for the prevention of chronic diseases in adult life.

## Figures and Tables

**Table 1:** Characteristics of study population according to Chinese famine exposure.

	All	Nonexposed	Fetal-Exposed	Infant-Exposed
Birth date		1962.10–1964.9	1959.10–1961.9	1956.10–1958.9
**Recruitment in 2002**				
N, %	18984	8163 (43.0)	4352 (22.9)	6469 (34.1)
Moderately exposed, %	40.7	37.8	45.8	41.0
Severely exposed, %	59.3	62.2	54.3	59.0
Age, years, mean (SD)	41.3 (2.6)	38.6 (0.6)	41.6 (0.6)	44.6 (0.6)
Female (%)	55.2	55.8	55.4	54.3
Height, cm, mean (SD)	160.9 (8.2)	161.2 (8.2)	161.0 (8.1)	160.4 (8.2) *
Weight, kg, mean (SD)	60.7 (10.8)	60.6 (10.8)	61.3 (10.9) *	60.4 (10.8)
WC, cm, mean (SD)	78.2 (9.5)	77.7 (9.4)	78.8 (9.6) *	78.5 (9.6) *
BMI, kg/m^2^, mean (SD)	23.4 (3.3)	23.2 (3.2)	23.6 (3.4) *	23.4 (3.4) *
Overweight (%)	38.2	36.3	41.3	38.4
Obesity (%)	8.9	8.2	9.7	9.4
Pre-central Obesity (%)	31.6	29.5	33.8	32.8
Central Obesity (%)	17.6	15.5	19.4	18.9
**Recruitment in 2012**				
N, %	16594	6493 (39.1)	4126 (24.9)	5975 (36.0)
Moderately exposed, %	50.8	49.5	55.5	49.0
Severely exposed, %	49.2	50.5	44.5	51.0
Age, years, mean (SD)	51.1 (2.7)	48.2 (0.9)	51.2 (0.9)	54.1 (1.0)
Female (%)	57.8	58.7	56.7	57.5
Height, cm, mean (SD)	160.4 (8.1)	160.7 (7.9)	160.5 (8.0)	160.0 (8.2) *
Weight, kg, mean (SD)	62.8 (10.7)	63.1 (10.7)	63.0 (10.7)	62.2 (10.7) *
WC, cm, mean (SD)	82.8 (9.8)	82.6 (9.8)	83.1 (9.7) *	82.7 (9.8)
BMI, kg/m^2^, mean (SD)	24.3 (3.4)	24.4 (3.4)	24.4 (3.4)	24.2 (3.4) *
Overweight (%)	51.3	51.8	52.1	50.3
Obesity (%)	13.7	14.5	13.8	12.8
Pre-central Obesity (%)	52.5	51.8	53.8	52.4
Central Obesity (%)	32.4	31.6	33.3	32.6

WC: Waist circumference. BMI: Body mass index. Overweight, systemic obesity, and central obesity were defined using the Chinese criteria for adults. Moderately exposed was defined as an excess death rate lower than 50.0%. * *p* < 0.05 (Bonferroni correction); statistical significance was compared with the nonexposed group (October 1962 to September 1964).

**Table 2:** Associations of famine exposure with central obesity risk in different severity of famine areas

	Nonexposed	Fetal-Exposed	Infant-Exposed
**Central Obesity in 2002**			
Waist circumference (cm)	77.7 (9.4)	78.8 (9.6)	78.5 (9.6)
Prevalence (%)	15.5	19.4	18.9
Odds ratio (95% CI)	1.00 (Ref)	1.31 (1.19–1.44)	1.28 (1.17–1.40)
*p*		<0.0001	<0.0001
*Stratified by famine severity*			
Moderately exposed			
Waist circumference (cm)	78.5 (9.6)	79.5 (9.9)	79.8 (9.9)
Prevalence (%)	18.0	22.4	23.2
Odds ratio (95% CI)	1.00 (Ref)	1.32 (1.14–1.52)	1.40 (1.23–1.60)
*p*		0.0002	0.0075
Severely exposed			
Waist circumference (cm)	77.1 (9.2)	78.2 (9.3)	77.5 (9.2)
Prevalence (%)	14.0	17.0	15.9
Odds ratio (95% CI)	1.00 (Ref)	1.24 (1.08–1.42)	1.17 (1.03–1.32)
*p*		0.0022	0.0139
**Central Obesity in 2012**			
Waist circumference (cm)	82.6 (9.8)	83.1 (9.7)	82.7 (9.8)
Prevalence (%)	31.6	33.3	32.6
Odds ratio (95% CI)	1.00 (Ref)	1.09 (1.00–1.19)	1.04 (0.97–1.13)
*p*		0.0493	0.2823
*Stratified by famine severity*			
Moderately exposed			
Waist circumference (cm)	82.9 (9.9)	83.2 (9.9)	82.9 (9.7)
Prevalence (%)	33.0	33.5	33.6
Odds ratio (95% CI)	1.00 (Ref)	1.03 (0.91–1.17)	1.02 (0.91–1.15)
*p*		0.6473	0.7145
Severely exposed			
Waist circumference (cm)	82.4 (9.7)	82.9 (9.5)	82.6 (9.8)
Prevalence (%)	30.6	33.2	31.9
Odds ratio (95% CI)	1.00 (Ref)	1.13 (1.01–1.27)	1.05 (0.95–1.16)
*p*		0.0331	0.3327

All odds ratios used the nonexposed group as the reference. Odds ratio (95% CI): adjusted for sex, residential areas, education level, marital status, household income, current drinking status, current smoking status, and physical activity.

**Table 3:** Associations of famine exposure with central obesity risk in different severity of famine areas: sensitivity analyses.

	Nonexposed	Fetal-Exposed	Infant-Exposed
**Central Obesity in 2002**			
**Sensitivity analyses A**			
Moderately exposed			
Prevalence (%)	17.0	20.4	20.9
Odds ratio (95% CI)	1.00 (Ref)	1.26 (1.13–1.41)	1.33 (1.20–1.47)
*p*		<0.0001	<0.0001
Severely exposed			
Prevalence (%)	11.7	15.0	11.3
Odds ratio (95% CI)	1.00 (Ref)	1.28 (1.01–1.62)	0.93 (0.75–1.16)
*p*		0.0411	0.5348
**Sensitivity analyses B**			
Moderately exposed			
Prevalence (%)	11.0	13.8	15.2
Odds ratio (95% CI)	1.00 (Ref)	1.26 (1.13–1.41)	1.33 (1.20–1.47)
*p*		<0.0001	<0.0001
Severely exposed			
Prevalence (%)	8.2	11.0	9.6
Odds ratio (95% CI)	1.00 (Ref)	1.28 (1.01–1.62)	0.93 (0.75–1.16)
*p*		0.0411	0.5348
**Central Obesity in 2012**			
**Sensitivity analyses A**			
Moderately exposed			
Prevalence (%)	32.3	32.7	32.9
Odds ratio (95% CI)	1.00 (Ref)	1.02 (0.93–1.12)	1.01 (0.93–1.10)
*p*		0.6629	0.8198
Severely exposed			
Prevalence (%)	29.5	35.8	31.7
Odds ratio (95% CI)	1.00 (Ref)	1.32 (1.11–1.58)	1.12 (0.96–1.31)
*p*		0.0017	0.1584
**Sensitivity analyses B**			
Moderately exposed			
Prevalence (%)	22.4	22.2	23.7
Odds ratio (95% CI)	1.00 (Ref)	0.98 (0.84–1.15)	1.06 (0.92–1.22)
*p*		0.8308	0.4090
Severely exposed			
Prevalence (%)	21.1	25.2	23.9
Odds ratio (95% CI)	1.00 (Ref)	1.27 (1.11–1.45)	1.17 (1.04–1.32)
*p*		0.0005	0.0102

Sensitivity analyses A: Defining severity of famine according to excess death rate 150%; Sensitivity analyses B: Excluding participants with BMI ≥ 28.0 kg/m^2^. All odds ratios used the nonexposed group as the reference. Odds ratio (95% CI): adjusted for sex, residential areas, education level, marital status, household income, current drinking status, current smoking status, and physical activity.
